# Numerical representations for flow velocity and shear rate inside electromagnetically levitated droplets in microgravity

**DOI:** 10.1038/s41526-019-0067-2

**Published:** 2019-03-25

**Authors:** Xiao Xiao, Jonghyun Lee, Robert W. Hyers, Douglas M. Matson

**Affiliations:** 1 Department of Mechanical Engineering, Tufts University, Medford, MA USA; 2 Department of Mechanical Engineering, Iowa State University, Ames, IA USA; 3 Department of Mechanical & Industrial Engineering, University of Massachusetts, Amherst, MA USA

## Abstract

Electromagnetic levitation techniques are used in a microgravity environment to allow materials research under containerless conditions while limiting the influence of gravity. The induced advective flow inside a levitated molten alloy droplet is a key factor affecting solidification phenomena while potentially influencing the measurement of thermophysical properties of metallic alloy. It is thus important to predict the flow velocity under various operation conditions during melt processing. In this work, a magnetohydrodynamic model is applied over the range of conditions under which electromagnetically levitated droplets are processed to represent the maximum flow velocity and shear rate as a polynomial function of heating voltage, density, viscosity, and electrical conductivity of molten materials. An example is given for the ternary steel alloy Fe-19Cr-21Ni (at%) to demonstrate how internal advection under different heater settings becomes a strong function of alloy temperature and is a determining factor in the transition from laminar to turbulent flow conditions. The results are directly applicable to a range of other materials with properties in the range considered, including Ni-based superalloys, Ti-6Al-4V, and many other commercially-important alloys.

## Introduction

Containerless processing techniques involving electromagnetic levitation (EML) provide the capability to position and process a highly reactive molten metal sample without use of a crucible while conducting thermophysical property measurements or solidification studies. For thermophysical property evaluations, the viscosity, density, surface tension, resistivity, and heat capacity of molten metal sample can be measured; for solidification studies the focus is on nucleation phenomena, growth mechanism, and phase selection. In either case, conditions may be significantly influenced or controlled by the advective flow inside the levitated molten metal droplet.^1^ For instance, the viscosity measurement of molten metals could be greatly affected by internal turbulent flow^2–5^ induced by the electromagnetic forces required to position, levitate and heat a sample, and well-controlled internal flow conditions are necessary to support the experiments; for phase selection in steels, the transformation of metastable to stable phases during rapid solidification is strongly affected by^6–9^ and could be controlled^10^ by applied advection inside the molten sample thus influencing development of the final microstructure.

For an EML facility, an alternating electromagnetic field is applied to a conductive sample located within a water-cooled coil as part of a high frequency oscillating circuit. Eddy currents induced inside the sample provide heating and positioning functions at different frequencies of the oscillating circuits, and the temperature of the sample is controlled by adjusting the heating control voltage. Meanwhile, the advective flow inside the molten sample is induced by the applied Lorenz force when the electromagnetic field is imposed, and velocity could be high under large heater setting, and turbulent flow may result. Okress et al.^11^ presented an analysis of heating power and electromagnetically levitated droplet, Szekely et al.^12–15^ developed the mutual inductance method to calculate electromagnetic forces in the spherical droplets, and Lohöfer^16–18^ developed an analytical model for the absorbed power, current distribution and impedance of an electromagnetically levitated metal sphere. Compared to the terrestrial environment, a microgravity environment provides the opportunity to maintain stable EML conditions with greatly reduced positioning forces. The levitated molten sample will form an approximately spherical shape and the induced flow inside the sample can achieve a wide range of flow velocity from laminar to turbulent conditions.^6,19^

Due to the difficulty of measurement of the flow inside the molten sample directly from experiment, numerical methods are utilized to simulate the advective flow field and predict related variables such as local flow velocity and shear rate inside the levitated molten metal droplets under given experimental parameters such as the sample’s physical properties and coil settings. For magnetohydrodynamic (MHD) simulation, in previous work by Szekely et al.^12,20^, MHD models for the electromagnetically levitated droplets was developed using a k–ε turbulence model for both terrestrial and microgravity environments. Recent work by Hyers et al.^1,4^ reported results for laminar flow in spherical droplets in a microgravity EML facility, and extended the results to turbulent flow of gravitationally-deformed droplets in ground-based EML. Berry et al.^3^ surveyed the turbulence models and stated that RNG k–ε turbulence model (Renormalization Group method variation) is the most appropriate model for EML droplets. Lee et al.^21^ validated the k–ε turbulence model through the comparison between the experiments and the predicted flow velocity along the surface of an electromagnetically levitated molten copper-cobalt droplet in the terrestrial environment which showed excellent agreement between model and experimental observations. The flow is usually characterized by the Reynolds number (Re) as defined in Eq. (1), which represents the ratio of inertial effects to viscous effects and indicates the laminar or turbulent condition of the flow.

1 $${\mathrm{Re}} = \frac{{\rho ud}}{\mu }$$

where *μ* is the viscosity, *ρ* is the density, *u* is the velocity, and *d* is the diameter of the sample droplet. For the laminar-turbulent transition that is characterized Reynolds number, Hyers et al.^22^ suggested that the transition occurs at Re around 500 to 600, which is experimentally observed from the formation and perturbation of the stagnation line at the equator of the droplet. Lee et al.^23,24^ also predicted the flow velocity of electromagnetically levitated iron-cobalt droplet in support of the experiments on board the International Space Station (ISS) with characteristic constraints of temperature and heating current appropriate to test conditions and determined the corresponding laminar and turbulent conditions related to the given geometry and realistic assumptions of the thermophysical properties of the alloy including density, viscosity, and electrical conductivity. Besides the k–ε turbulence models, Bojarevics et al.^25,26^ used pseudospectral methods to solve the Navier–Stokes equations with k–ω turbulence model, Ai^27^ used direct numerical simulation of turbulent flow in EML.

In the present work, the model development is based on microgravity EML using a superposition levitation method (the coil configuration is called SUPOS for “superposition”) on board ISS; the design specifications of ISS-EML SUPOS coil are described by Lohöfer.^28,29^ MHD simulations using laminar model and RNG k–ε turbulence model are conducted to predict the flow velocity and shear rate inside a molten droplet when electromagnetically levitated by the SUPOS coil in a microgravity environment in both the laminar and turbulent regime, as a function of a series of key experimental parameters. For a given sample size, these parameters include heating control voltage of the coil, density, viscosity, and electrical conductivity of the sample material. Finally, the results from MHD simulation are represented as polynomial expressions for convenient reference to be applied to molten materials that requires characterization by MHD methods; in practice this involves defining key material properties as a function of temperature such that the flow field becomes a function of applied heating control voltage and sample temperature, only.

## Results

### General model

The MHD simulation is performed for a 6.5 mm electromagnetically levitated droplet in microgravity with the ISS-EML SUPOS coil under fixed positioning control voltage $U_{\mathrm{ctr}}^P$ at 5.21 V, and multiple conditions of heating control voltage, density, viscosity, and electrical conductivity which are shown in Table 1. For a general levitated molten droplet, as expansion plus fitting of monographs in,^30^ the flow velocity and shear rate are predicted and represented as function of heating control voltage, density, viscosity, and electrical conductivities based on around 10,000 discrete modelling runs for both of laminar and turbulent models.

**Table 1:** Operation conditions for ISS-EML Levitated Droplet

Parameters	Values
Heating control voltage (V)	$U_{\mathrm{ctr}}^H = 0.01-6.00 \ \left( {8 \ \,{\mathrm{levels}}} \right)$
Density (kg m^−3^)	*ρ* = 5000–10,000 (11 levels)
Viscosity (Pa s)	*μ* = 0.001–0.040 (8 levels)
Electrical conductivity (S m^−1^)	*σ*_*e*,*l*_ = 2.0 × 10–6.0 × 10^6^ (7 levels)

The model is solved in axisymmetric two-dimensional space. *u*_*θ*_ and *u*_*r*_ denote the flow velocity in the angular and radial coordinate respectively, *u* is the velocity magnitude, and *u*_max_ is the the maximum flow velocity. $\dot \gamma$ denotes the magnitude of shear rate inside the droplet as defined in Eq. (2), and $\dot \gamma _{max}$ is the maximum shear rate in the flow field.

2 $$\dot \gamma = \left| {r\frac{\partial }{{\partial r}}\left( {\frac{{u_\theta }}{r}} \right) + \frac{1}{r}\frac{{\partial u_r}}{{\partial \theta }}} \right|$$

At each electrical conductivity value, the maximum velocity *u*_max_ and maximum shear rate $\dot \gamma _{max}$ are fitted into third degree polynomials with four variables over a representative range of heating control voltage $U_{\mathrm{ctr}}^H$ (*i*), density *ρ* (*j*), natural logarithm of viscosity *ln μ* (*k*), and natural logarithm of electrical conductivity *ln* *σ*_*e*,*l*_ (*s*), as presented in Eq. (3), where the coefficients *p*_*ijks*_ are derived using least-squares approach from the raw data. The quality of the fits for the interpolated maximum velocity $\hat u_{max}$ and interpolated maximum shear rate $\widehat {\dot \gamma }_{max}$ are evaluated using *R*-squared metric, where the value closer to 1.0 means a better fit has been obtained.

3 $$\begin{array}{*{20}{l}} {\hat u_{\max }\,or \ \, \widehat {\dot \gamma }_{max}} \hfill & = \hfill & {\mathop {\sum}\limits_{i,j,k,s} {p_{ijks}U_{\mathrm{ctr}}^H} \,{}^{i}\rho ^{j}(ln\,\mu )^{k}(ln\,\sigma _{e,l})^s} \hfill \\ {R- \mathrm{squared}} \hfill & = \hfill & {1 - \frac{{\sum (\hat u_{\mathrm{max}} \,-\, u_{\mathrm{max}})^2}}{{\sum (\hat u_{\mathrm{max}} \,-\, \overline {u_{\mathrm{max}}} )^2}}\,or\,1 - \frac{{\sum \left( {\widehat {\dot \gamma }_{\mathrm{max}} \,-\, \dot \gamma _{\mathrm{max}}} \right)^2}}{{\sum \left( {\widehat {\dot \gamma }_{\mathrm{max}} \,-\, \overline {\dot \gamma _{\mathrm{max}}} } \right)^2}}} \hfill \end{array}$$

To evaluate the contribution of each term to the overall fit, the absolute value of Pearson correlation coefficient (PCC), as defined in Eq. (4), is calculated between simulation results *Y* = *u*_max_ or $\dot \gamma _{max}$ for each term $X_{ijks} = U_{\mathrm{ctr}}^H\,{}^i\rho ^j(ln\,\mu )^k(ln\,\sigma _{e,l})^s$.

4 $$\left| {\rho _{X_{ijks},Y}} \right| = \left| {\frac{{\mathrm{cov}(X_{ijks},Y)}}{{\sigma _{X_{ijks}}\sigma _Y}}} \right|$$

**Fig. 1:**
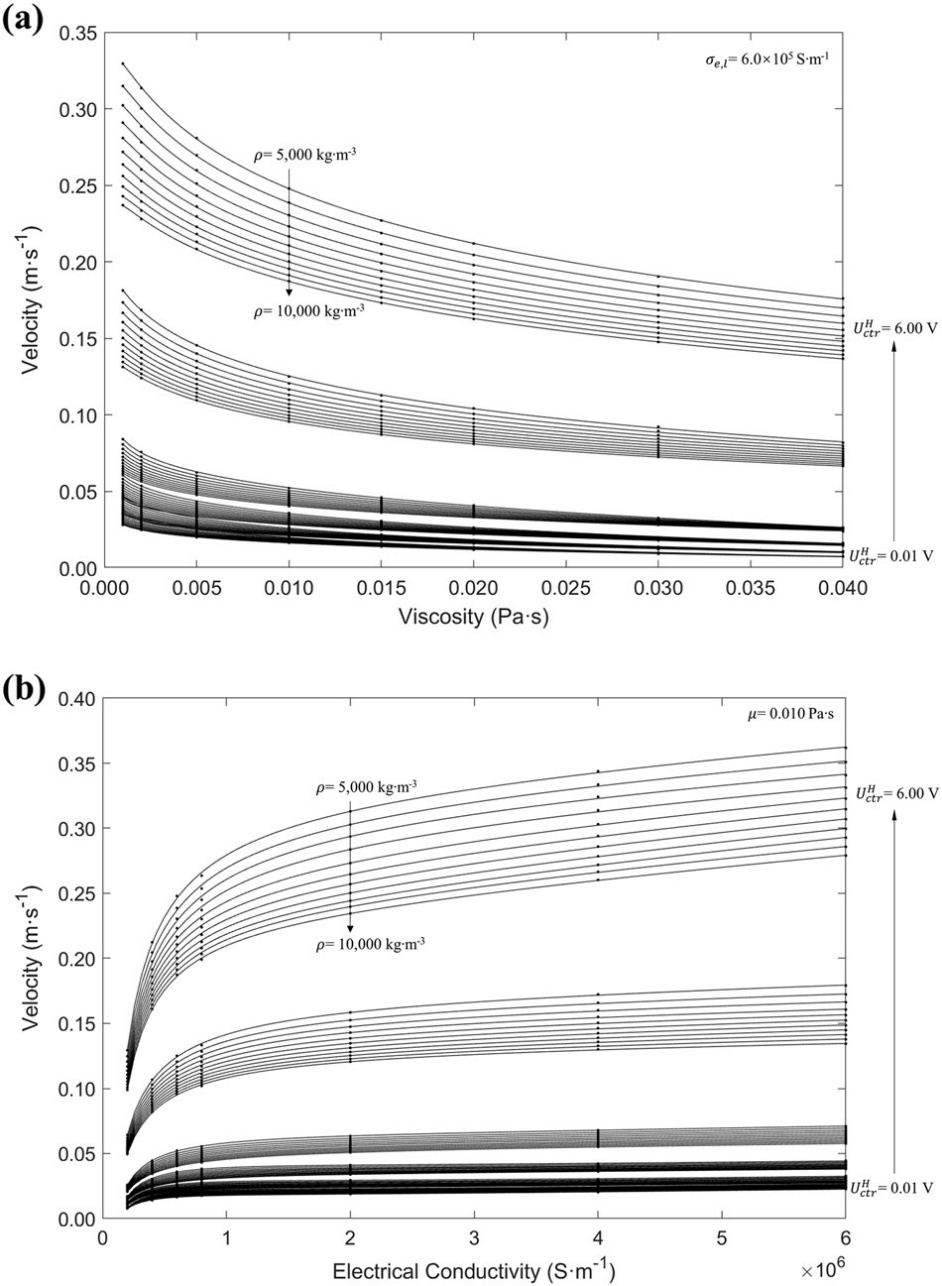
Maximum Velocity as a function of Heating Control Voltage, Density, Viscosity, and Electrical Conductivity (each figure includes six groups of curves where $U_{\mathrm{ctr}}^H$ is valued at 0.01, 0.20, 0.50, 1.0, 3.0, and 6.0 V, and each group with the same $U_{\mathrm{ctr}}^H$ contains 11 curves where *ρ* ranges from 5000 to 10,000 kg m^−3^ for step size of 500). **a** Maximum velocity at *σ*_*e*,*l*_ = 6.0 × 10^5^ S m^−1^, **b** maximum velocity at *μ* = 0.010 Pa s

The value of $\left| {\rho _{X_{ijks},Y}} \right|$ is between 0 and 1 for positive correlation, where a value closer to 1.0 means a signification correlation; cov(*X*_*ijks*_, *Y*) is the covariance between *X*_*ijks*_ and *Y*, and *σ* are their standard deviation. To select the dominating terms *X*_*ijks*_ and reduce the dimension of the regression equation, *X*_*ijks*_ is ordered by the value $\rho _{X_{ijks},Y}$, and the first *N* terms of *X*_*ijks*_ are included in the *N*th regression testing until R-squared increases to value closer to 1.0 and converges. The regression tests show that the first 21 terms were significant, as displayed in Table 2. The fitted coefficients *p*_*ijks*_ and overall *R*-squared values using laminar and turbulent models are displayed separately, and using these values the predicted maximum velocity $\hat u_{\mathrm{max}}$ and predicted maximum shear rate $\widehat {\dot \gamma }_{\mathrm{max}}$ can be readily estimated for any combination of parameters of $U_{\mathrm{ctr}}^H$, *ρ*, *μ*, and *σ*_*e*,*l*_, by using Eq. (3) with all the coefficients *p*_*ijks*_ presented in Table 2 and related indices *i*, *j*, *k*, *s* applied to each term. Figure 1a shows an example of the predicted $\hat u_{\mathrm{max}}$ as function of viscosity *μ*, heating control voltage $U_{\mathrm{ctr}}^H$ and density *ρ* under electrical conductivity *σ*_*e*,*l*_ = 6.0 × 10^5^ S m^−1^, and Fig. 1b shows $\hat u_{\mathrm{max}}$ as function of *σ*_*e*,*l*_, $U_{\mathrm{ctr}}^H$, and *ρ* under *μ* = 0.010 Pa s.

**Table 2:** Polynomial coefficients of maximum velocity and shear rate for ISS-EML Levitated Molten Droplet

	Laminar model		Turbulent model	
	Velocity (m s^−1^)	Shear rate (s^−1^)	Velocity (m s^−1^)	Shear rate (s^−1^)
*p*_*0000*_	2.705 × 10^−1^	3.801 × 10^2^	1.025 × 10^−1^	2.213 × 10^2^
*p*_*0001*_	−2.375 × 10^−2^	−3.601 × 10^1^	−9.377 × 10^−3^	−1.796 × 10^1^
*p*_*0010*_	1.481 × 10^−1^	1.152 × 10^2^	9.369 × 10^−2^	1.432 × 10^2^
*p*_*0011*_	−1.758 × 10^−2^	−1.196 × 10^1^	−1.221 × 10^−2^	−1.662 × 10^1^
*p*_*0012*_	3.930 × 10^−4^	3.746 × 10^−2^	3.322 × 10^−4^	4.002 × 10^−1^
*p*_*0021*_	−1.402 × 10^−5^	−1.304 × 10^−1^	−2.197 × 10^−5^	4.452 × 10^−2^
*p*_*1000*_	−9.354 × 10^−1^	−1.634 × 10^3^	−4.259 × 10^−1^	−7.906 × 10^2^
*p*_*1001*_	1.100 × 10^−1^	1.881 × 10^2^	5.454 × 10^−2^	8.993 × 10^1^
*p*_*1002*_	−3.587 × 10^−3^	−5.873	−1.796 × 10^−3^	−2.927
*p*_*1010*_	−3.068 × 10^−2^	−8.963 × 10^1^	−1.167 × 10^−3^	−1.992 × 10^1^
*p*_*1011*_	−1.638 × 10^−3^	1.088 × 10^−2^	−1.233 × 10^−3^	−2.826
*p*_*1020*_	−2.927 × 10^−3^	−5.758	−7.224 × 10^−4^	−3.369
*p*_*1100*_	9.942 × 10^−6^	1.262 × 10^−2^	4.693 × 10^−6^	1.031 × 10^−2^
*p*_*1101*_	−8.575 × 10^−7^	−1.170 × 10^−3^	−4.767 × 10^−7^	−9.095 × 10^−4^
*p*_*1110*_	1.135 × 10^−6^	1.635 × 10^−3^	5.908 × 10^−7^	1.245 × 10^−3^
*p*_*1200*_	2.931 × 10^−10^	4.580 × 10^−7^	1.775 × 10^−10^	3.035 × 10^−7^
*p*_*2000*_	3.861 × 10^−3^	6.457	−9.398 × 10^−4^	1.933
*p*_*2001*_	7.098 × 10^−4^	7.534 × 10^−1^	3.206 × 10^−4^	6.064 × 10^−1^
*p*_*2010*_	1.269 × 10^−3^	1.993	2.329 × 10^−4^	9.055 × 10^−1^
*p*_*2100*_	−7.639 × 10^−8^	−9.026 × 10^−5^	−1.077 × 10^−8^	3.413 × 10^−6^
*p*_*3000*_	−6.038 × 10^−4^	−4.668 × 10^−1^	−1.623 × 10^−4^	−4.845 × 10^−1^
*R*−*squared*	0.9963	0.9939	0.9982	0.9957

## Discussion

In the current settings, the heating field produces much stronger flow than the positioner field for most of the common operating range. The magnitude of positioner-induced flow and correlated shear rate slightly increases with the positioner voltage $U_{\mathrm{ctr}}^P$ in the range from 2.0 to 10.0 V, where ${\mathrm{d}}\hat u_{\mathrm{max}}/{\mathrm{d}}U_{\mathrm{ctr}}^P$ is <0.0002 m s^−1^ V^−1^ and ${\mathrm{d}}\widehat {\dot \gamma }_{\mathrm{max}}{\mathrm{/}}\mathrm{{d}}U_{\mathrm{ctr}}^P$ is <0.8 s^−1^ V^−1^. The variance induced from different positioner voltage $U_{\mathrm{ctr}}^P = 2.0\,{\mathrm{V}}$ to 10.0 V is <0.001 m s^−1^ for $\hat u_{\mathrm{max}}$ and <4.0 s^−1^ for $\widehat {\dot \gamma }_{\mathrm{max}}$ compared to the results with $U_{\mathrm{ctr}}^P = 5.21\,{\mathrm{V}}$, presented up to 10% error when the heater is minimized, and up to 3% error when $U_{\mathrm{ctr}}^H$ is >0.2 V. This variation with positioner is negligible for most operational conditions, so positioner voltage $U_{\mathrm{ctr}}^P$ is excluded from the fits.

The droplet dimension is an additional factor in the MHD model which was studied previously^1,24^ that the maximum velocity $\hat u_{\mathrm{max}}$ and maximum shear rate $\widehat {\dot \gamma }_{\mathrm{max}}$ increases for larger droplet diameter *d*, and gives basis for an extrapolation formula presented in Eq. (5) for *d* = 5.0 mm−7.0 mm based on the predictions under *d*_*0* _ = 6.5 mm.

5 $$\frac{{\left. {\hat u_{\mathrm{max}}} \right|_d}}{{\left. {\hat u_{\mathrm{max}}} \right|_{d_0}}}\,or\,\frac{{\left. {\widehat {\dot \gamma }_{\mathrm{max}}} \right|_d}}{{\left. {\widehat {\dot \gamma }_{\mathrm{max}}} \right|_{d_0}}} \approx \frac{{0.253\,d^2 - 1.887\,d-3.393}}{{0.253\,d_0^2 - 1.887\,d_0-3.393}}$$

### Practical application to a specific case

The general model provides coefficients that are used to predict flow at a given heater setting for a given density, viscosity, and electrical conductivity. In practice, an experimentalist would know these thermophysical properties for a particular sample material as a function of temperature, and thus flow can be predicted given the heater setting and temperature. Then the predictions can be used either as a forecasting tool before a test is run or as a characterization tool based on the observed pyrometer temperatures after a test is run. The approach is to select a temperature at a given heater setting, evaluate the thermophysical properties, and generate a plot of the flow velocity and shear rate over the available experiment control-space.

For an application of the general model, the ternary steel alloy Fe-19Cr-21Ni (atomic %) was selected to represent the family of industrially-cast austenitic alloys for phase selection experiments in microgravity on-board the ISS. To quantify advection during these tests, MHD modeling was conducted over the range of conditions accessible using the ISS-EML SUPOS coil. Conditions would be selected such that the 6.5 mm diameter molten sample droplet could achieve a wide range of heating rates (up to d*T/*d*t* = 200 K s^−1^ at *T*_*m*_) or cooling rates (d*T/*d*t* = 0–50 K s^−1^ at *T*_*m*_ in vacuum or d*T/*d*t* = 0–100 K s^−1^ at *T*_*m*_ in helium) and a broad range of thermal hold temperatures *T* = *T*_*m*_ ± 200 K such that each is characterized by distinct quasistatic flow conditions depending on the heating control voltage. The thermophysical propriety values vary with the temperature as shown in Table 3.

**Table 3:** Baseline material properties for Fe-19Cr-21Ni (at.%)

Properties	Values (*T*_*m*_ = 1715 K)
Density (kg m^−3^)	*ρ* = −0.71∙*T* + 8209^2^
Viscosity (Pa s)	*μ* = exp(11,980/*T* − 11.54)^5^
Electrical conductivity (S m^−1^)	*σ*_*e*,*l*_ = 6.63 × 10^5^ + 380(*T* − *T*_*m*_)^1,31^

For operation conditions heating control voltage $U_{\mathrm{ctr}}^H = 0.01\,{\mathrm{V}}-5.7\,{\mathrm{V}}$ with the positioner maintained at $U_{\mathrm{ctr}}^P = 5.21\,{\mathrm{V}}$, and temperatures over the range *T* = 1515 K–1915 K (*T*_*m*_ − 200 K to *T*_*m*_ + 200 K), the MHD model was utilized to predict the advective flow field and local shear rate inside the 6.5 mm molten Fe-19Cr-21Ni droplet.

**Fig. 2:**
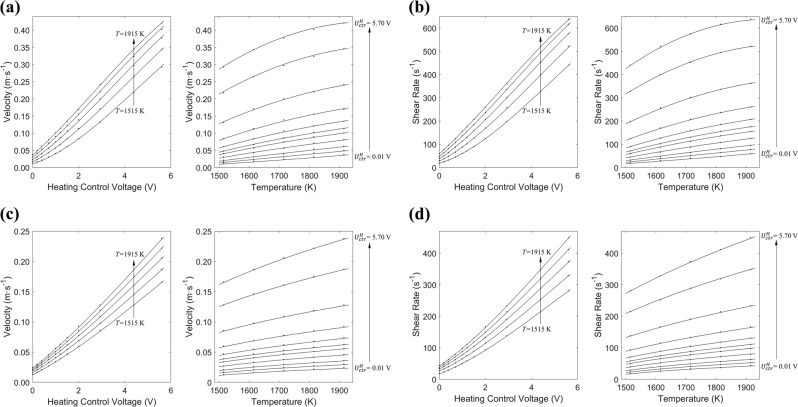
Fe-19Cr-21Ni Maximum Velocity and Maximum Shear Rate as a function of Heating Control Voltage and Temperature (dots represent the results from the general model where $U_{\mathrm{ctr}}^H$ is valued at 0.01, 0.20, 0.40, 0.70, 1.00, 1.20, 1.50, 2.90, 4.40, and 5.70 V, and *T* ranges from 1515 to 1915 K for step size of 100). **a** Laminar model: maximum velocity, **b** Laminar model: maximum shear rate, **c** Turbulent model: maximum velocity, **d** Turbulent model: maximum shear rate

Figure 2 shows the predicted maximum velocity $\hat u_{\mathrm{max}}$ and predicted maximum shear rate $\widehat {\dot \gamma }_{\mathrm{max}}$ of Fe-19Cr-21Ni under various heating control voltages $U_{\mathrm{ctr}}^H$ and temperatures *T* with both of laminar and turbulence models, where the dots represent the results from the general model extrapolated from Eq. (2) and Table 2, and the curves represent the correlated predicted values as defined in Eq. (6) and Table 4, which are further fitted to obtain expressions of $\hat u_{\mathrm{max}}$ and $\widehat {\dot \gamma }_{\mathrm{max}}$ as function of $U_{\mathrm{ctr}}^H$ and *T*, based on the extrapolated values from the general model.

6 $$\hat u_{\mathrm{max}}\,\mathrm {or}\ \,\widehat {\dot \gamma }_{\mathrm{max}} = \mathop {\sum }\limits_{i,j} p_{ij}U_{\mathrm{ctr}}^H\,{}^iT^j$$

**Table 4:** Polynomial coefficients of maximum velocity and maximum shear rate for ISS-EML Levitated Fe-19Cr-21Ni Droplet

	Laminar model		Turbulent model	
	Velocity (m s^−1^)	Shear rate (s^−1^)	Velocity (m s^−1^)	Shear rate (s^−1^)
*p*_*00*_	−8.675 × 10^−2^	−1.376 × 10^2^	−2.943 × 10^−2^	−7.501 × 10^1^
*p*_*01*_	6.468 × 10^−5^	1.025 × 10^−1^	2.819 × 10^−5^	6.190 × 10^−2^
*p*_*10*_	−4.051 × 10^−1^	−6.804 × 10^2^	−7.681 × 10^−2^	−1.761 × 10^+2^
*p*_*11*_	4.286 × 10^−4^	7.247 × 10^−1^	9.342 × 10^−5^	1.967 × 10^−1^
*p*_*12*_	−9.704 × 10^−8^	−1.686 × 10^−4^	−1.965 × 10^−8^	−4.102 × 10^−5^
*p*_*20*_	2.246 × 10^−2^	3.418 × 10^+1^	1.820 × 10^−3^	3.519
*p*_*21*_	−9.066 × 10^−6^	−1.376 × 10^−2^	1.730 × 10^−7^	1.351 × 10^−3^
*p*_*30*_	−6.403 × 10^−4^	−9.437 × 10^−1^	−1.308 × 10^−4^	−3.905 × 10^−1^
*R-squared*	0.9998	0.9999	0.9999	0.9999

**Fig. 3:**
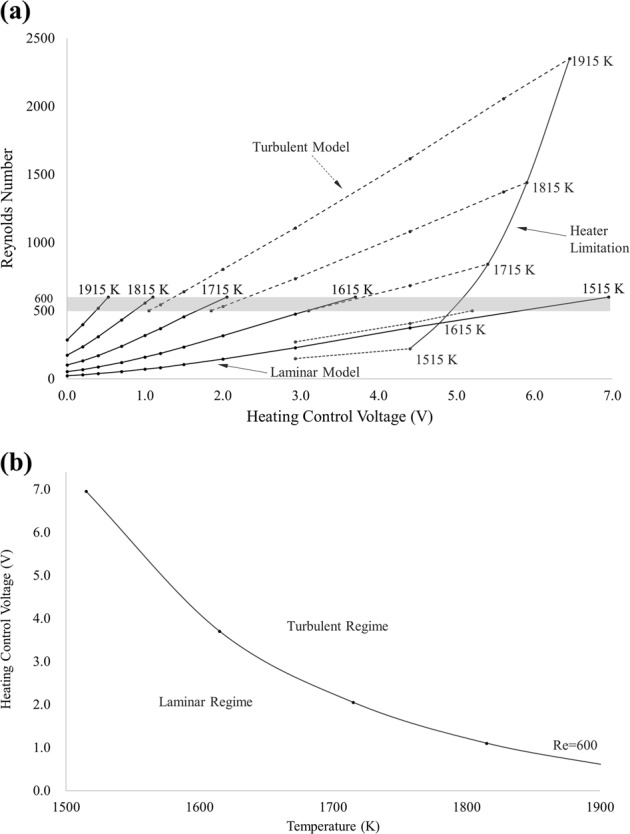
Fe-19Cr-21Ni Reynolds number and flow conditions. **a** Reynolds Number as function of heating control voltage and temperature, **b** critical heating control voltage and temperature

Based on the Reynolds number calculated using Eq. (1) correlated to the predicted maximum velocity, the flow conditions are determined to be either laminar, transitional, or turbulent. Figure 3a shows the Reynolds number over a range of heating control voltage and temperature, utilizing both laminar and turbulent models. On the figure, an upper temperature limit is shown representing the heater setting to achieve an isothermal hold. This limit is critical for planning of conditions to conduct thermophysical property measurement at a desired temperature and for identifying the heating control limit for undercooling experiments.

The laminar flow starts to become unsteady at Re = 500 and becomes turbulent above Re = 600.^22^ For the accessible range of conditions, the turbulent flow is transitional and not fully-developed nor isotropic, in part due to the constraints on eddy size imposed by the finite size of the droplet. It is appropriate to use the results from laminar model to calculate the Reynolds number that determines the flow conditions. A critical combination of the heating control voltage and temperature can be derived such that the correlated Reynolds number is larger than 600 in the range above the critical values. In Fig. 3b, the critical heating control voltage can be seen to vary with the temperature. Above the curve the flow condition is turbulent and below the curve is laminar. This provides a criterion for determination and selection of flow regimes for planning of experimental conditions.

In conclusion, the velocity and shear rate inside electromagnetically levitated droplet in microgravity with the ISS-EML SUPOS coil is numerically predicted and represented using a previously-validated MHD model. For a levitated molten droplet of arbitrary material properties, the flow is represented as function of heating control voltage, density, viscosity, electrical conductivity, and droplet dimensions, for convenient reference over a wide range of possible metallic materials. As an example of how these results may be applied, the ternary steel alloy Fe-19Cr-21Ni system was selected such that the key material properties all become a function of temperature only. The maximum flow velocity is then represented as functions of heating control voltage and temperature; the critical combination of heating voltage and temperature is provided to predict the flow conditions determining the laminar or turbulent condition of the internal advective flow.

## Methods

### ISS-EML SUPOS coil specification

For the experiment conducted in microgravity onboard the ISS, the sample of 5.0–7.0 mm in diameter was positioned and heated using ISS-EML SUPOS coil^28^ in vacuum or in 350 mbar inert helium or argon gas. The ISS-EML SUPOS coil is a single-coil/dual-current type with upper and lower coils wound in one piece such that a single system is used for both heating and positioning. The alternating current through the coil runs at a frequency of 150 kHz for the positioner and generates a quadrupole electromagnetic force field to locate the sample near the center of the coil set. The heating current runs at 350 kHz and generates a dipole electromagnetic field that controls the sample temperature through a balance between the resistive heating due to the eddy currents and heat loss to the environment due to conduction and radiation. The coil currents and the control voltage has the following linear relations, where $I_0^H$ and $I_0^P$ are the heating and positioning current, $U_{\mathrm{ctr}}^H$ and $U_{\mathrm{ctr}}^P$ are the heating and positioning control voltage of the facility.

7 $$\begin{array}{l}I_0^H = 19.09 + 19.00 \cdot U_{\mathrm{ctr}}^H\\ I_0^P = 27.21 + 27.21 \cdot U_{\mathrm{ctr}}^P\end{array}$$

### MHD modeling techniques

MHD of the EML droplet consists interaction between electromagnetic field through the conductive molten liquid and the internal flow induced from the electromagnetic forces.^23^ The electromagnetic forces in the molten alloy droplet induced from the EML coil could be calculated through solving a reduced form of quasi-stationary Maxwell’s equations,^18^ which is defined in Eq. (8),

8 $$\begin{array}{l}\nabla \cdot {\mathbf{B}} = 0\\ \nabla \times {\mathbf{E}} = - \frac{{\partial {\mathbf{B}}}}{{\partial {\mathrm{t}}}}\\ \nabla \times {\mathbf{H}} = {\mathbf{J}}\end{array}$$

where **J** is the induced current, **H** is the magnetic field, **B** is the magnetic flux density, and **E** is the electric field. The electromagnetic force which is also known as Lorentz force is written as,

9 $${\mathbf{F}} = {\mathbf{J}} \times {\mathbf{B}}$$

The method of mutual inductances^12^ is used to numerically solve reduced Maxwell’s equations and calculate the electromagnetic force, utilizing a subroutine developed separately.^1^ Because the magnetic Reynolds number is so small, the coupling between electromagnetism and flow is one-way: the magnetic field drives the flow, but is not significantly perturbed by the flow.

The internal flow could be assumed as incompressible and viscous, which is governed by the Navier–Stokes equations,

10 $$\begin{array}{l}\nabla \cdot {\mathbf{u}} = 0\\ \frac{{\partial {\mathbf{u}}}}{{\partial {\mathrm{t}}}} + {\mathbf{u}} \cdot \nabla {\mathbf{u}} =\frac{1}{\rho }\left(- \nabla p + {\mu }\nabla ^2{\mathbf{u}} + {\mathbf{F}}\right)\end{array}$$

where **u** is the velocity vector, *p* is the pressure, *μ* and *ρ* is the viscosity and density, and **F** is the momentum source which corresponds to the electromagnetic force per unit volume for the EML.

The boundary conditions are assumed to be a slip wall, where there is no shear stress on the free surface, and no flux across the surface,

11 $$\begin{array}{l}\left. {{\mathbf{\tau }} \cdot {\mathbf{i}}_t} \right|_{r = 1} \,=\, 0\\ \left. {u_r} \right|_{r = 1} \,=\, 0\end{array}$$

where **τ** is shear stress, **i**_*t*_ is the tangent unit vector, and *u*_*r*_ is the radial component of *u*.

For simulation of turbulent flow, the RNG *k*–*ε* turbulence model is adopted. Adding extra terms, the vector of turbulent velocity *u* consists of the time-averaged velocity ${\bar{\mathbf u}}$ and the fluctuation **u**′,

12 $$\begin{array}{l}{\mathbf{u}} = {\bar{\mathbf u}} + {\mathbf{u}}^\prime \\ {\bar{\mathbf u}} = \mathop {{\lim }}\limits_{T \to \infty } \frac{1}{T}\mathop {\int }\nolimits_0^T {\mathbf{u}}\,\mathrm{d}t\end{array}$$

Eq. (10) then becomes the time-averaged Navier–Stokes equations,

13 $$\frac{{\partial {\bar{\mathbf u}}}}{{\partial t}} + {\bar{\mathbf u}} \cdot \nabla {\bar{\mathbf u}} = \frac{1}{\rho }\left(- \nabla \bar p + {\mu }\nabla ^2{\bar{\mathbf u}} + {\mathbf{F}}\right) - \nabla \cdot \left( {\overline {{\mathbf{u}}^\prime {\mathbf{u}}^\prime } } \right)\\$$

where $\bar p$ is the averaged pressure, and $\overline {{\mathbf{u}}\prime {\mathbf{u}}\prime }$ is the Reynolds stress term describing the additional stresses generated from turbulent fluctuations.

Two additional equations, the turbulent kinetic energy equation and energy dissipation equation, are included in the *k*-*ε* turbulence model, which represent the dissipation rate of the turbulent kinetic energy,

14 $$\begin{array}{*{20}{l}} {\frac{{\partial k}}{{\partial t}} + {\bar{\mathbf u}} \cdot \nabla k} \hfill & = \hfill & {\left( {{\mathbf{u}} + \frac{{u_t}}{{\sigma _k}}} \right)\nabla ^2k + P_k - \varepsilon } \hfill \\ {\frac{{\partial \varepsilon }}{{\partial t}} + {\bar{\mathbf u}} \cdot \nabla \varepsilon } \hfill & = \hfill & {\left( {\frac{{\mathrm{\mu }}}{\rho } + \frac{{u_t}}{{\sigma _\varepsilon }}} \right)\nabla ^2\varepsilon + C_{1\varepsilon }\frac{\varepsilon }{k}P_k - C_{2\varepsilon }\frac{{\varepsilon ^2}}{k}} \hfill \end{array}$$

with additional boundary conditions,

15 $$\begin{array}{l}\left. {\frac{{\partial k}}{{\partial r}}} \right|_{r = 1} \,=\, 0\\ \left. {\frac{{\partial \varepsilon }}{{\partial r}}} \right|_{r = 1} \,=\, 0\end{array}$$

The turbulent kinetic energy is defined as $k = \frac{1}{2}\overline {u_i^\prime u_i^\prime }$, $P_k = \tau _{i.j}\left( {\partial \bar u_i/\partial x_j} \right)$ is the kinetic energy production, *u*_*t*_ = *C*_*μ*_(*k*^2^/*ε*) is the kinematic eddy viscosity, and ${\mathrm{\varepsilon }} = \frac{{\mathrm{\mu }}}{\rho }\frac{{\partial \overline {{\mathrm{u}}_i^\prime } }}{{\partial x_j}}\frac{{\partial \overline {{\mathrm{u}}_i^\prime } }}{{\partial x_j}}$ is the dissipation rate.

In Eq. (14), the RNG *k*–*ε* model uses the following coefficients,^32^

16 $$\begin{array}{*{20}{l}} {C_{1\varepsilon }} \hfill & = \hfill & {1.42} \hfill \\ {C_{2\varepsilon }} \hfill & = \hfill & {1.68} \hfill \\ {C_\mu } \hfill & = \hfill & {0.085} \hfill \\ {\sigma _k} \hfill & = \hfill & {0.72} \hfill \\ {\sigma _\varepsilon } \hfill & = \hfill & {0.72} \hfill \end{array}$$

**Fig. 4:**
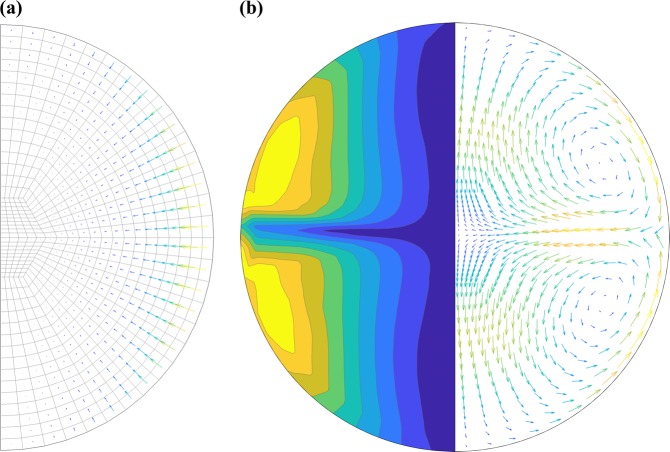
MHD Model for Electromagnetically Levitated Droplet. **a** Mesh grid with interpolated electromagnetic force density superimposed. **b** Contour of shear rate magnitude (left side); Vectors of flow velocity (right side)

In the MHD model, the sample is assumed to be at the center of the coil with limited translational oscillations, is of spherical shape with limited surface deformation and at thermal pseudo-steady state with constant and homogeneous thermophysical properties. In practice, the variance due to oscillation and surface deformation may introduce error <8%, and that of thermal equilibrium is negligible. The steady-state solver of the prescribed MHD model is based on a finite volume method through the commercial package ANSYS Fluent. The model includes a mesh consisting of an optimized number of 550 cells and 591 nodes as shown in Fig. 4a, superimposed with the electromagnetic force as the momentum source term in the shape of arrows.

For the heater-dominated MHD simulation results, the flow typically consists of two toroidal circulation loops near the stagnation line at the equator of the droplet, turning inward the sphere where the electromagnetic force archives a maximum around the equator. The predicted flow patterns are displayed as a vector plot of flow velocity and contour of shear rate magnitude as shown in Fig. 4b on right and left side respectively. For the flow with relatively low Reynolds number below 500, the laminar model is appropriate and accurate; for Reynolds numbers much larger than 600 the flow is turbulent and the results from the RNG *k*–*ε* turbulence model are more appropriate.

Note that the analysis may not be appropriate for application to experimental conditions during rapid heating—for example during melting the sample experiences surface oscillations and inhomogeneous temperatures across sample; during short pulse applications that used to induce surface oscillations for property evaluations, even if deformations are small, the flow is transient and not quasistatic as required by the present model. Future work will extend the model to allow predictions of the shape of deformed samples under either transient or quasi-static conditions.

### Reporting summary

Further information on experimental design is available in the Nature Research Reporting Summary linked to this article.

## Supplementary information

Nomenclature

Reporting Summary

## Data Availability

The data that support the findings of this study are available from the corresponding author upon request.

## Supplementary information

**Supplementary information** accompanies the paper on the *npj Microgravity* website (10.1038/s41526-019-0067-2).
