# Supplementary material for: Numerical representations for flow velocity and shear rate inside electromagnetically levitated droplets in microgravity
Source: NPJ Microgravity. 2019 Mar 25;5:7. doi: 10.1038/s41526-019-0067-2 (PMC6433928; doi:10.1038/s41526-019-0067-2)
Supplement: Supplementary file 1 — Nomenclature [file 41526_2019_67_MOESM1_ESM.docx]

**Supplemental Materials**

**Nomenclature**

__________________________________________________________________________

T (K) Temperature

T_m_ (K) Melting temperature

$d$ (mm) Diameter of sample droplet

$\rho$ (kg∙m^-3^) Density

$\mu$ (Pa∙s) Viscosity

$\sigma_{e,l}$ (S∙m^-1^) Electrical conductivity

$U_{ctr}^{H}$ (V) DC Heating control voltage

$U_{ctr}^{P}$ (V) DC Positioning control voltage

$I_{0}^{H}$ (A) AC Heating current

$I_{0}^{P}$ (A) AC Positioning current

$u$ (m∙s^-1^) Magnitude of flow velocity

$u_{\theta}$ (m∙s^-1^) Angular velocity

$u_{r}$ (m∙s^-1^) Radial velocity

$u_{max}$ (m∙s^-1^) Maximum flow velocity

$\dot{\gamma}$ (s^-1^) Magnitude of shear rate

$\dot{\gamma}_{max}$ (s^-1^) Maximum shear rate

$k$ (m^2^∙s^-1^) Turbulent kinetic energy

$\varepsilon$ (m^2^∙s^-3^) Turbulent dissipation rate

$\mathbf{u}$ (m∙s^-1^) Velocity vector

$\mathbf{u}^{\mathbf{'}}$ (m∙s^-1^) Fluctuation velocity

$\boldsymbol{\tau}$ (N∙m^-2^) Shear stress

$\mathbf{F}$ (N∙m^-1^) Lorentz force

$\mathbf{H}$ (A∙m^-1^) Magnetic field

$\mathbf{B}$ (T) Magnetic flux density

$\mathbf{J}$ (A) Induced current

__________________________________________________________________________
